# Supplementary material for: Spatial variation in red deer density in a transboundary forest ecosystem
Source: Sci Rep. 2023 Mar 20;13:4561. doi: 10.1038/s41598-023-31283-7 (PMC10027870; doi:10.1038/s41598-023-31283-7)
Supplement: Supplementary file 1 — Supplementary Tables. [file 41598_2023_31283_MOESM1_ESM.pdf]

## Supplementary Information

Tourani, M. et al. Spatial variation in red deer density in a transboundary forest ecosystem.

Model definition can be found here: `https://github.com/MTourani/OpenRepo/tree/master/BohemianCervus`

**Table S1:** Overview of the microsatellite markers used for individual identification of red deer *Cervus elaphus* samples in this study. The allele numbers represent the numbers of different alleles present in the population under study. The marker AMELXY was used for sex determination.

| Marker | Length (bp) | Number of alleles | Reference |
|---|---|---|---|
| IDVGA55 | 195 - 209 | 11 | [1] |
| BMC1009 | 282 - 306 | 14 | [1] |
| TGLA53 | 151 - 193 | 18 | [1] |
| BM203 | 214 - 236 | 19 | [1] |
| CSSM16 | 152 - 162 | 6 | [2] |
| CSSM19 | 139 - 169 | 14 | [2] |
| Haut14 | 108 - 148 | 16 | [2] |
| ETH225 | 132 - 170 | 15 | [2] |
| AMELXY | 228 - 282 | 2 | [3] |

**Table S3:** Total, jurisdiction-specific, and sex-specific estimates of red deer *Cervus elaphus* density and abundance with 95% credible interval in parentheses. BFNP: Bavarian Forest National Park, Germany; SFNR: Bavarian State Forest Neureichenau, Germany; SNP: Šumava National Park, the Czech Republic.

| | Abundance (95% CI) | | | Density (95% CI) | | |
|---|---|---|---|---|---|---|
| **Region** | **Female** | **Male** | **Total** | **Female** | **Male** | **Total** |
| **SNP** | 990.4 (840 - 1166) | 1061.6 (919 - 1230) | 2052 (1836 - 2292) | 1.7 (1.4 - 2) | 1.8 (1.6 - 2.1) | 3.5 (3.1 - 3.9) |
| **BFNP** | 369.5 (287 - 473) | 286.4 (220 - 370) | 655.9 (547 - 786) | 1.5 (1.2 - 2) | 1.2 (0.9 - 1.5) | 2.7 (2.2 - 3.2) |
| **SFNR** | 46.6 (25 - 80) | 97 (68 - 136) | 143.6 (105 - 193) | 0.3 (0.2 - 0.5) | 0.7 (0.5 - 0.9) | 1 (0.7 - 1.3) |
| **Total** | 1406.4 (1229 - 1612) | 1445 (1288 - 1626) | 2851.4 (2609 - 3119) | 1.4 (1.3 - 1.6) | 1.5 (1.3 - 1.7) | 2.9 (2.7 - 3.2) |

**Table S2:** Genotyping error rates, i.e., allelic dropout (ADO) and false alleles (FA), as well as percentage of positive polymerase chain reaction (PCR) of genotyped red deer *Cervus elaphus* faecal samples collected in the Bohemian Forest Ecosystem. ADO and FA rates are denoted as percentage of all positive PCRs per microsatellite locus, as well as weighted average over all loci. $H_{exp}$ stands for expected heterozygosity and $H_{obs}$ denotes the heterozygosity observed in the data set.

| **Microsatellite** | **ADO (%)*** | **FA (%)** | **%PCR** | **$H_{exp}$ (%)** | **$H_{obs}$ (%)** |
|---|---|---|---|---|---|
| Haut14 | 2.9 | 0.8 | 96 | 74 | 73 |
| CSSM16 | 3.6 | 0.8 | 98 | 77 | 78 |
| BM203 | 5.6 | 1.1 | 88 | 85 | 74 |
| BMC1009 | 5.8 | 0.0 | 91 | 79 | 73 |
| IDVGA55 | 2.9 | 0.4 | 98 | 83 | 83 |
| CSSM19 | 3.3 | 1.1 | 82 | 78 | 68 |
| TGLA53 | 4.4 | 1.0 | 95 | 86 | 85 |
| ETH225 | 5.3 | 1.7 | 95 | 90 | 87 |
| Mean | 4.2 | 0.9 | 93 | 82 | 78 |

* For estimation of ADO, only heterozygote loci are used for all microsatellites, because ADO cannot be detected at homozygote loci.

**Table S4:** Covariate effects on red deer *Cervus elaphus* density including protected (i.e., non-intervention zones; $\beta_{\mathrm{SNPp}}$ and $\beta_{\mathrm{BFNPp}}$) and unprotected regions ($\beta_{\mathrm{SFNR}}$ and $\beta_{\mathrm{BFNP}}$), elevation ($\beta_{\mathrm{E}}$), proportion of disturbance ($\beta_{\mathrm{R}}$), and their interaction effect ($\beta_{\mathrm{ER}}$).

| | | **Female** | | | **Male** | |
|---|---|---|---|---|---|---|
| **Coefficients** | **Mean** | **Lower 95% CI** | **Upper 95% CI** | **Mean** | **Lower 95% CI** | **Upper 95% CI** |
| $\beta_{\mathbf{BFNP}}$ | –2.8 | –4.8 | –1.4 | –2.2 | –3.8 | –1 |
| $\beta_{\mathbf{BFNPp}}$ | 0.2 | –0.3 | 0.6 | 0.02 | –0.5 | 0.5 |
| $\beta_{\mathbf{SFNR}}$ | –1.9 | –2.7 | –1.1 | –1 | –1.5 | –0.5 |
| $\beta_{\mathbf{SNPp}}$ | –0.4 | –1 | 0.1 | 0.1 | –0.4 | 0.5 |
| $\beta_{\mathbf{E}}$ | 0.03 | –0.2 | 0.3 | –0.3 | –0.5 | –0.1 |
| $\beta_{\mathbf{R}}$ | 0.7 | –0.5 | 1.8 | –1.9 | –3.4 | –0.4 |
| $\beta_{\mathbf{ER}}$ | 0.42 | –0.3 | 1.1 | 2.1 | 1.3 | 2.8 |

# References


[1] Valière, N. *et al.* Importance of a pilot study for non-invasive genetic sampling: genotyping errors and population size estimation in red deer. *Conservation Genetics* **8**, 69–78 (2007).

[2] Frantz, A. *et al.* Genetic structure and assignment tests demonstrate illegal translocation of red deer (*Cervus elaphus*) into a continuous population. *Molecular Ecology* **15**, 3191–3203 (2006).

[3] Gurgul, A., Radko, A. & Słota, E. Characteristics of X- and Y-chromosome specific regions of the amelogenin gene and a PCR-based method for sex identification in red deer (*Cervus elaphus*). *Molecular Biology Reports* **37**, 2915–2918 (2010).
